# Evaluation of Different Power of Near Addition in Two Different Multifocal Intraocular Lenses

**DOI:** 10.1155/2016/1395302

**Published:** 2016-06-01

**Authors:** Ugur Unsal, Gonen Baser

**Affiliations:** ^1^Batigoz Eye Health Center, 35210 Izmir, Turkey; ^2^Department of Ophthalmology, Egepol Hospital, 35270 Izmir, Turkey

## Abstract

*Purpose.* To compare near, intermediate, and distance vision and quality of vision, when refractive rotational multifocal intraocular lenses with 3.0 diopters or diffractive multifocal intraocular lenses with 2.5 diopters near addition are implanted.* Methods.* 41 eyes of 41 patients in whom rotational +3.0 diopters near addition IOLs were implanted and 30 eyes of 30 patients in whom diffractive +2.5 diopters near addition IOLs were implanted after cataract surgery were reviewed. Uncorrected and corrected distance visual acuity, intermediate visual acuity, near visual acuity, and patient satisfaction were evaluated 6 months later.* Results*. The corrected and uncorrected distance visual acuity were the same between both groups (*p* = 0.50 and *p* = 0.509, resp.). The uncorrected intermediate and corrected intermediate and near vision acuities were better in the +2.5 near vision added intraocular lens implanted group (*p* = 0.049, *p* = 0.005, and *p* = 0.001, resp.) and the uncorrected near vision acuity was better in the +3.0 near vision added intraocular lens implanted group (*p* = 0.001). The patient satisfactions of both groups were similar.* Conclusion*. The +2.5 diopters near addition could be a better choice in younger patients with more distance and intermediate visual requirements (driving, outdoor activities), whereas the + 3.0 diopters should be considered for patients with more near vision correction (reading).

## 1. Introduction

Multifocal intraocular lenses (IOLs) were designed to correct distance and near vision, and their effectiveness in achieving this has been demonstrated before [1]. Most multifocal IOLs are refractive or diffractive in design. In contrast to refractive IOLs, diffractive IOLs are independent from the pupil diameter and provide a better near vision but demonstrate more disturbances in night vision [2]. The classic designs can cause side effects, including loss of contrast sensitivity [3], halos and dysphotopsia, and patient dissatisfaction [4, 5]. Because success with these IOLs requires high patient motivation, innovative designs have been developed in an attempt to address the side effects.

Multifocal IOLs are based on the optical principle of creating two focal planes, one for distance vision and the other for near vision [6, 7]. AcrySof® IQ ReSTOR® (Alcon Laboratories, Inc., Fort Worth, TX, USA) IOLs combine an apodized diffractive zone (for distance and near or intermediate vision) and refractive zone (for distance vision) to enhance visual acuity at multiple distances and direct light to near or distant focal points, according to lighting conditions and pupil diameter [7, 8]. The ReSTOR +3.0 D and +2.5 D multifocal IOLs provide effective near, intermediate, and distance vision, with the ReSTOR +2.5 D IOL having a near focal point shifted toward intermediate distances and demonstrating lesser glare and dysphotopsia [6–8].

A new IOL, the multifocal Lentis Mplus LS-313 (Oculentis GmbH), has a rotationally asymmetric design with a sectorial-embedded near zone. The IOL also splits the image into two focal planes [9]. Theoretically, fewer optical side effects of the new IOL should be expected because there are no ring segments in the optic for refraction or diffraction and the embedded near sector reflects unwanted side images away from the retina [10].

The aim of this study was to evaluate and compare the visual results achieved in patients, in whom rotational multifocal (Lentis Mplus MF30) and diffractive multifocal (AcrySof ReSTOR) intraocular lenses were implanted after cataract surgery. To avoid confusion, the AcrySof SV25TO group will be named as ReSTOR group and the Lentis-313 MF30 group will be named as Oculentis group.

## 2. Methods

This retrospective study was adhered to the Declaration of Helsinki. Informed consent has been obtained from all patients enrolled between January 2010 and January 2012. The study cohort comprised presbyopic ametropic patients who had cataracts or were not suitable candidates for laser vision correction. 41 eyes of 41 patients in whom rotational +3.0 D near addition IOLs are implanted and 30 eyes of 30 patients who underwent phacoemulsification cataract surgery in whom diffractive +2.5 D near addition IOLs are implanted were enrolled into the study. The choice of the IOL depended on the age and psychosocial and physical status of the patients. This means that in patients who were active workers and had more outdoor hobbies the +2.5 D addition was preferred.

Exclusion criteria were a history of glaucoma or retinal detachment, corneal disease, corneal surgery, ocular inflammation, neuroophthalmic disease, macular degeneration or retinopathy, and keratometric cylinder greater than 1.00 diopter (D). The inclusion of amblyopic patients was restricted to those with a corrected distance visual acuity (CDVA) of 6/9 or better in the amblyopic eye and of 6/6 or better in the fellow eye.

### 2.1. Patient Assessment

All patients had a preoperative examination that included autorefraction and tonometry (Topcon Co., Ltd.), corneal topography (Pentacam, Oculus, Inc.), uncorrected distance visual acuity (UDVA), CDVA, uncorrected near visual acuity (UNVA), endothelial cell count (SP2000P specular microscope, Topcon Europe BV), biometry (IOLMaster, Carl Zeiss Meditec AG), subjective and cycloplegic refractions, slit-lamp evaluation, and dilated fundoscopy. Visual acuity was measured at distance and near vision with a Snellen visual acuity chart and converted to logMAR scores. Data (axial length (AL), anterior chamber depth, and keratometry) from the IOLMaster were used for IOL calculation. All eyes were targeted for emmetropia. Uncorrected distance visual acuity and corrected distance visual acuity (UDVA and CDVA), uncorrected intermediate visual acuity and distance-corrected intermediate visual acuity (UIVA and CIVA), uncorrected near visual acuity and distance-corrected near visual acuity (UNVA and CNVA), and patient satisfaction were evaluated 6 months later. The type of IOL was randomized, but the visual necessities of the patients were taken into consideration. The patient satisfaction was evaluated with a questionnaire. The near vision addition was +1.0 in the ReSTOR patients, who needed aid for reading. There was no near vision addition necessary in the Oculentis group.

### 2.2. Intraocular Lenses

Two different IOLs, multifocal Lentis Mplus LS-313MF30 (Oculentis GmbH) with +3.0 D near vision correction and AcrySof IQ ReSTOR SV25T0 (Alcon Laboratories, Inc., Fort Worth, TX, USA) with +2.5 D near vision correction, are used.

### 2.3. Surgical Technique

The surgeries have been performed by two surgeons (Mehmet Soyler and Ugur Unsal). The pupil was dilated with cyclopentolate. Oxybuprocaine 1.0% and tetracaine 1.0% local anesthesia drops were applied.

Lens extraction was performed using a standard phacoemulsification technique through a 2.2 mm incision. All incisions were made on the steepest corneal meridian to neutralize corneal astigmatism. After phacoemulsification, a foldable Mplus IOL was inserted in the capsular bag through a 2.75 mm corneal incision using the Viscoject 2.2 injector (Viscoject 2.2, Cartridge-Set LP604240M, Oculentis GmbH) or a Monarch IOL injector was used to implant the AcrySof IQ ReSTOR IOLs. Surgery in the second eye was usually performed 1 week later. Postoperatively, patients were instructed to instill 1 drop of moxifloxacin 0.5% (Vigamox) 4 times daily for 2 weeks, 1 drop of dexamethasone 0.1% (Maxidex) 4 times daily for 2 weeks, and 1 drop of ketorolac trometamol 0.5% (Acular) 4 times daily for 1 month.

### 2.4. Statistical Analysis

Statistical analyses were performed using the RStudio software version 0.98.501 via R language. The variables were investigated using visual (histograms, probability plots) and analytical methods (Kolmogorov-Smirnov/Shapiro-Wilk test) to determine whether or not they are normally distributed. Descriptive analyses were presented using means and standard deviations for normally distributed variables (preoperative and postoperative UDVA, CDVA, UIVA, CIVA, UNVA, and CNVA). The variables showed a normal distribution (*p* > 0.05), so an independent *t*-test was used to compare the continuous variables between the groups. A value of *p* < 0.05 was considered statistically significant.

## 3. Results

The Oculentis implanted group consisted of 41 eyes of 41 patients. The cohort comprised 20 men (48.78%) and 21 women (51.22%). The mean age of the patients at the time of surgery was 59.4 ± 7.9 (20 to 84 years). The mean power of implanted IOLs was 22.5 ± 5.0 D (range from 6.0 to 34.5 D).

The ReSTOR implanted group consisted of 30 eyes of 30 patients. The cohort comprised 16 men (53.44%) and 14 women (46.66%). The mean age of the patients at the time of surgery was 56.4 ± 8.3 (21 to 77 years). The mean power of implanted IOLs was 22.0 ± 4.5 D (range from 7.0 to 33.5 D).

The preoperative refractive and UCVA values were not different between both groups (*p* = 0.251 and *p* = 0.322, resp.). The CDVA and UDVA scores were not different between both groups either (*p* = 0.50 and *p* = 0.509, resp.).

The postoperative UIVA, CIVA, and CNVA scores were better in the ReSTOR implanted patients (*p* = 0.049, *p* = 0.005, and *p* = 0.001, resp.), whereas the UNVA scores were better in the Oculentis implanted group (*p* = 0.001).

In general, the satisfactions of the subjects of both groups were similar. Both cohorts declared that they would recommend this procedure to others with a high percentage (99%). The glare and halo complaints were 9.9% in the Oculentis group and 10.4% in the ReSTOR group.

The results are summarized in Table 1.

## 4. Discussion

The visual requirements of cataract patients are different. These requirements depend on age, business, sociocultural status, and hobbies. Finding the convenient IOL can be a challenge in patients with high expectations [11]. The present study indicates that, for monocular and binocular vision, CIVA and UIVA were significantly better in patients with a diffractive multifocal IOL with an addition power of +2.5 D, whereas UNVA was better with an addition of 3.0 D.

Regarding near vision, Oculentis in the current study provided a mean CNVA of 0.098 (logMAR), which was within the range of the reported values of Schmickler et al. and García-Domene et al. [12, 13]. Alió et al. reported better near vision with the Oculentis than accommodative IOLs [14].

The ReSTOR diffractive IOL with +2.5 near vision addition is merely designed for people who are more active in working life and especially driving to the ability to read the dashboard. These people depend more on distance and intermediate visual acuity [15]. On the other hand, this amount of correction could bring difficulties in near vision, which is more necessary for reading. We observed in our study a better IVA in patients in the ReSTOR group. Besides this, the CNVA scores were better with the ReSTOR also. We observed no disturbances in patients with +1.00 D near vision correction.

A thorough meta-analysis of multifocal IOLs was performed by Cochener et al. comparing literature on different types of multifocal IOLs published since 2000. The study found the mean UDVA of all multifocal IOLs to be 0.093 logMAR. When analyzing different types of multifocal IOLs, the mean UDVA was 0.105 logMAR for diffractive IOLs, 0.085 logMAR for refractive IOLs, and 0.067 logMAR when the ReSTOR IOL (Alcon Laboratories, Inc.) was analyzed separately [16].

Previous studies compared the performance of the Lentis Mplus IOL with that of some commonly used multifocal IOLs. Alió et al. compared the Lentis Mplus LS-312 MF30 with the Acri.Lisa 366D (Zeiss) diffractive IOL. Intermediate vision and contrast sensitivity were better with the Lentis Mplus. However, the Acri.Lisa provided better distance and near visual outcomes [17]. Van der Linden et al. compared the Lentis Mplus LS-312 MF30 with the ReSTOR SN6AD1 IOL 8 (+3.0 D). They found that the IOLs achieved comparable distance vision, while the ReSTOR provided better near visual acuity [18]. The ReSTOR SN6AD1 IOL has a near addition of +3.0 D. Therefore a better near vision can be expected. A similar comparison of the Lentis Mplus and the ReSTOR IOL was performed by Alfonso et al. and Alió et al. Both studies found that near vision was better with the ReSTOR IOL. Intermediate vision was better with the Lentis Mplus in one study and was not different from that with the ReSTOR IOL in the other study [17, 19].

The main advantage of the asymmetric design of the Lentis Mplus IOL over traditional rotationally symmetrical multifocal IOLs is the presence of only 1 transition zone between the aspheric distance vision zone and the inferior sector-shaped near vision zone. This technology should in theory reduce the source of scattering and aberrations, minimize halos and glare, and improve contrast sensitivity [19].

To our knowledge, this is the first study in the literature in which +2.50 and +3.0 added multifocal IOLs are compared. The patients' satisfactions in our groups were high and similar. The glare and halo complaints were tolerable. The ReSTOR patients who needed +1.0 addition for near sight were not unhappy, because they reported that they use these glasses only for special situations such as reading medicine prospects or sewing. The ReSTOR IOL was enough for more routine near activities like newspaper reading or working with computers. Our opinion is that the correct patient choice was here the key factor for the happiness of both patient groups.

In conclusion, we believe that the correct patient selection and IOL decision are the key factors in successful cataract surgery. The Alcon ReSTOR +2.5 near addition could be a better choice in younger patients with more distance and intermediate visual requirements. However, Oculentis could be considered for patients who are more interested in reading with a more nonactive lifestyle. Further investigations for the ideal intraocular lens are still present and it seems that it will take some time to invent the ideal one.

## Figures and Tables

**Table 1:** Results and comparison between the +2.5 D ReSTOR and +3.0 D Oculentis group.

Variables	Group	*N*	Mean	Std. deviation	Std. error mean	*p* value
Preoperative refraction	Oculentis	41	−1.896	4.8	0.8	0.251
	ReSTOR	30	−0.925	2.0	0.4	

Preoperative UDVA	Oculentis	41	0.624	0.2	0.0	0.322
	ReSTOR	30	0.690	0.3	0.1	

Postoperative refraction	Oculentis	41	−0.860	0.3	0.1	***0.001*** ^*∗*^
	ReSTOR	30	0.058	0.3	0.0	

Postoperative UDVA	Oculentis	41	0.056	0.2	0.0	0.500
	ReSTOR	30	0.033	0.1	0.0	

Postoperative UNVA	Oculentis	41	0.098	0.1	0.0	***0.001*** ^*∗*^
	ReSTOR	30	0.587	0.1	0.0	

Postoperative UIVA	Oculentis	41	0.312	0.0	0.0	***0.049*** ^*∗*^
	ReSTOR	30	0.287	0.1	0.0	

Postoperative CDVA	Oculentis	41	0.002	0.0	0.0	0.509
	ReSTOR	30	0.007	0.0	0.0	

Postoperative CNVA	Oculentis	41	0.210	0.0	0.0	***0.001*** ^*∗*^
	ReSTOR	30	0.153	0.1	0.0	

Postoperative CIVA	Oculentis	41	0.315	0.0	0.0	***0.005*** ^*∗*^
	ReSTOR	30	0.277	0.1	0.0	

^*∗*^ *p* < 0.05 is statistically significant.
